# Three-dimensional topology optimization model to simulate the external shapes of bone

**DOI:** 10.1371/journal.pcbi.1009043

**Published:** 2021-06-16

**Authors:** Misaki Sakashita, Shintaro Yamasaki, Kentaro Yaji, Atsushi Kawamoto, Shigeru Kondo

**Affiliations:** 1 Graduate School of Frontier Biosciences, Osaka University, Suita, Japan; 2 Graduate School of Engineering, Osaka University, Suita, Japan; 3 Toyota Central R&D Labs., Inc., Nagakute, Japan; Oxford, UNITED KINGDOM

## Abstract

Elucidation of the mechanism by which the shape of bones is formed is essential for understanding vertebrate development. Bones support the body of vertebrates by withstanding external loads, such as those imposed by gravity and muscle tension. Many studies have reported that bone formation varies in response to external loads. An increased external load induces bone synthesis, whereas a decreased external load induces bone resorption. This relationship led to the hypothesis that bone shape adapts to external load. In fact, by simulating this relationship through topology optimization, the internal trabecular structure of bones can be successfully reproduced, thereby facilitating the study of bone diseases. In contrast, there have been few attempts to simulate the external structure of bones, which determines vertebrate morphology. However, the external shape of bones may be reproduced through topology optimization because cells of the same type form both the internal and external structures of bones. Here, we constructed a three-dimensional topology optimization model to attempt the reproduction of the external shape of teleost vertebrae. In teleosts, the internal structure of the vertebral bodies is invariable, exhibiting an hourglass shape, whereas the lateral structure supporting the internal structure differs among species. Based on the anatomical observations, we applied different external loads to the hourglass-shaped part. The simulations produced a variety of three-dimensional structures, some of which exhibited several structural features similar to those of actual teleost vertebrae. In addition, by adjusting the geometric parameters, such as the width of the hourglass shape, we reproduced the variation in the teleost vertebrae shapes. These results suggest that a simulation using topology optimization can successfully reproduce the external shapes of teleost vertebrae. By applying our topology optimization model to various bones of vertebrates, we can understand how the external shape of bones adapts to external loads.

## Author summary

In this paper, we developed a computational method to investigate the relationship between three-dimensional bone shape and external loads imposed on bones. Many studies report that bone formation varies in response to external loads. An increased external load induces bone synthesis, whereas a decreased external load induces bone resorption. This relationship led to the hypothesis that the shape of bones adapts to external load. However, it remains unclear whether this hypothesis can explain the shape of bones. Here, we constructed a three-dimensional mathematical model that imitates the cellular activities of bone formation to attempt the reproduction of the shape of teleost vertebrae. In teleosts, the shape of the vertebrae differs among the species. We set the multiple types of external load conditions in the simulations and compared the simulation results with different teleost vertebrae. The produced structures that can resist the deformation of the surrounding tissues exhibited multiple structural features similar to the vertebrae of several teleost species. This result shows that the formation of bone shape can be explained by the adaptation to external load.

## Introduction

Elucidating how the shape of bones is formed is essential to obtain an in-depth understanding of vertebrate development, as the body shape of vertebrates depends on a skeleton that is composed of differently shaped bones. To maintain the body shape of vertebrates, bones must be able to withstand various continual external loads, such as those imposed by gravity and muscle tension. Previous studies have demonstrated that a change in these external loads causes a morphological change in bones. For instance, femur bones become thinner and sparser in the zero-gravity environment of space flight [1, 2]. In addition, immobility due to a spinal cord injury changes the cross-sectional tibial geometry from the typical teardrop appearance to a more circular shape [3], and a bipedal goat without forelegs exhibits a narrowing of the pelvis relative to that of quadrupedal goats [4]. These findings suggest that unlike the shape of organs that is mainly determined genetically, bone shape is influenced by the external loads.

Indeed, this possibility has been supported by cytological studies showing that the cellular activities involved in bone formation are controlled by external loads. The main types of cells involved in bone shape are osteoblasts and osteoclasts. Osteoblasts secrete and mineralize the bone matrix, whereas osteoclasts break down and resorb the bones. These opposing activities of osteoblasts and osteoclasts vary according to the external load condition through osteocytic or non-osteocytic regulation mechanisms [5–10]; an increased external load induces bone synthesis [11], whereas a decreased external load induces bone resorption [12, 13]. These findings support Roux’s paradigm that local cellular activities induced by external loads form bone shape [14–17] (sometimes this paradigm is integrated with other paradigm known as “Wolff’s law” [16, 18, 19]).

To confirm this paradigm, computer simulations of the cellular activities in bone formation have been performed. In the field of biomechanics, Huiskes and colleagues developed a bone remodeling algorithm in which bone density is regulated by the strain energy density [20–23]. In this bone remodeling algorithm, bone addition occurs in high-strain-energy density areas and bone removal occurs in low-strain-energy density areas, thereby imitating the activities of osteoblasts and osteoclasts that are regulated according to external loads. Accordingly, this algorithm could produce the pillar structure aligned to the direction of external loads, which is the characteristic of the bone internal structure formed by trabeculae [23, 24]. In the same period, many researchers have applied structural optimization methods to reproduce bone shape from an engineering perspective [25, 26]. One of the most widely utilized methods for this purpose is topology optimization [27–33]. Topology optimization optimizes the distribution of a material’s density according to a physical quantity such as strain and stress, and has been used to generate the optimal shapes of different types of structures, such as machine parts and architecture [34, 35]. Although topology optimization was initially developed for purposes not related to imitating the bone formation process, the fundamental of its algorithm is the same as the bone remodeling algorithm [36]. In stiffness maximization, the addition and removal of a material occur according to the strain energy density, effectively reproducing the internal structure of the bone. These computer simulations in the different fields have explained that the internal structure of the bone adapts to the external loads, thereby facilitating research on abnormal bone shapes due to diseases [37, 38].

In contrast to the extensive simulations of bone internal structures, few computer simulations have been performed to reproduce the external shape formed by the cortical bones. However, because both the internal and external structures of bones are formed by osteoblasts and osteoclasts, it is reasonable to hypothesize that the bone’s external shape can also be reproduced through the computer simulations. Based on this hypothesis, several researchers have attempted to reproduce the outline of the femur, tibia, and vertebrae as cross-sections [39–42]. Additionally, Mittag et al. [43, 44] produced three-dimensional (3D) tube-like structures to reproduce the external shape of the long bone shafts. However, actual bones exhibit 3D external shapes with a complex curved surface, which are difficult to represent with a cross-section or a simple geometric shape. Therefore, the aforementioned hypothesis remains to be examined. For this reason, we aimed to reproduce the 3D external shapes of bones. As topology optimization can be used to generate a variety of 3D structures, we considered that this approach would be suitable for simulating the 3D external shapes of bones. Furthermore, topology optimization can produce an optimal structure for multiple objectives, allowing for further modification of the simulation model. Hence, we used the stiffness maximization algorithm with topology optimization for the reproduction of external bone shapes.

**Fig 1:**
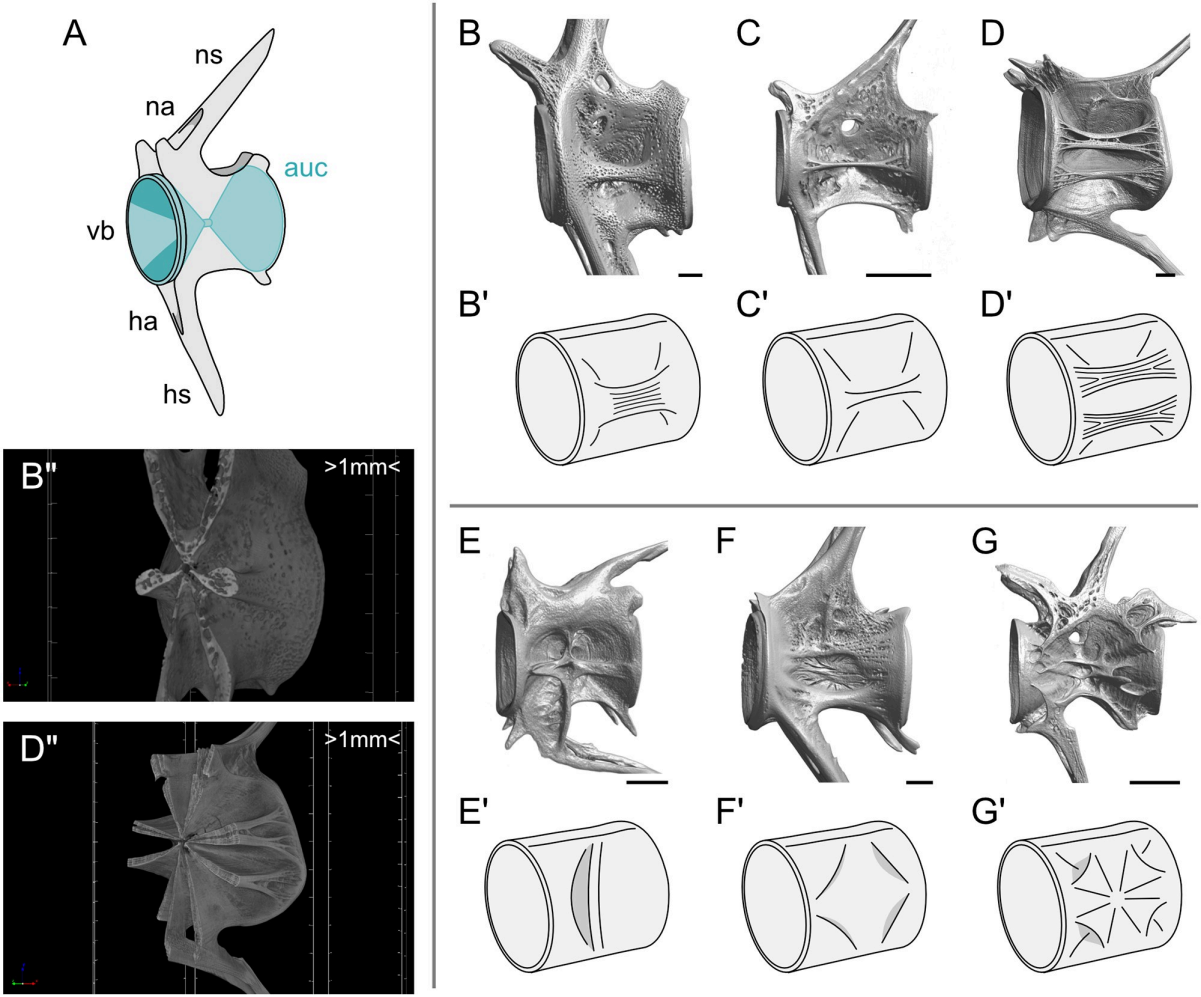
Anatomy of fish vertebrae. (A) Structure of the fish vertebrae. Abbreviations: auc: autocentrum; ns: neural spine; na: neural arch; vb: vertebral body; ha: hemal arch; hs: hemal spine. (B–G) Volume-rendered micro-computed tomography (micro-CT) images of left lateral views of the caudal vertebral bodies of (B) *Pagrus major*, (C) *Acropoma hanedai*, (D) *Zenopsis nebulosa*, (E) *Muraenesox cinereus*, (F) *Scarus forsteni*, and (G) *Macroramphosus sagifue*. (B’–G’) Schematic illustrations of the morphological features of (B–G) that displays (B) a single thick longitudinal plate-like ridge (thick trabecula), (C) a single thin longitudinal plate-like ridge, (D) two longitudinal plate-like ridges, (E) a transverse plate-like ridge, (F) hump-like structures rising on the edge of the vertebral body, (G) tarp-like triangle ridges extending from the center to the edge of the vertebral body. (B” and D”) Transverse sections at the midpoint of the vertebral bodies of (B”) *P. major* and (D”) *Z. nebulosa*. These images indicate that the lateral ridges extend from the vertebral body center. Scale bars: (B–F) 1 mm, and (G) 500 μm. In (B” and D”), the interval between scale markers is 1 mm.

In this study, we selected the teleost vertebrae as the subject for the simulation based on two advantageous characteristics. First, the skeleton of fish does not need to counteract gravity, unlike the case for terrestrial vertebrates. For this reason, the external load condition of teleost vertebrae is relatively simple. In a simulation, it is important to first set the load cases that define the type of external load that is applied to the bone, because the optimization process depends on the strain energy density caused by the load cases. As it is nearly impossible to directly examine the external load condition of bones, we need to try numerous optimizations for different load cases to reproduce the external shape of bones. Using teleost vertebrae allows us to apply this approach, as we can simply set the load cases. Second, the external shape of teleost vertebral bodies greatly varies among species. The vertebral bodies of teleost fish are mainly formed by the internal autocentrum and external arcocentrum, each with a distinct composition [45]. The internal autocentrum has an amphicoelous hourglass shape, which is invariable among species [46, 47] (Fig 1A). In contrast, the lateral structure of the vertebral bodies formed by the external arcocentrum varies among species [46–49] (Fig 1). This variation can be compared to the multiple 3D structures that are produced by optimizations for different load cases.

Here, we constructed a topology optimization model to attempt the reproduction of the lateral structures of teleost vertebral bodies. Because the external loads on teleost vertebrae arise from the deformation of the surrounding tissues [50, 51], we set multiple load cases based on anatomical studies and produced various types of structures. By comparing the optimization results to the teleost vertebrae, we examined whether the external shape of the teleost vertebrae can be reproduced using topology optimization.

## Mathematical modeling

### Topology optimization

In our topology optimization model, vertebral morphology is visualized as the distribution of the material density at each location *ρ*(**x**) of an analysis domain. **x** indicates a position in the analysis domain and *ρ*(**x**) ∈ [0, 1] is a continuous and relative density. *ρ* = 0 indicates that the region is void and *ρ* = 1 indicates that the region is full of material. To obtain the distribution of *ρ*(**x**), we solved the following stiffness maximization problem with the assumption of static, linear elastic behavior:

$$\begin{array}{c} \begin{array}{ccc} \underset{\rho (x)}{\text{minimize}} & f(\rho ) & \equiv \int_{\Gamma_{t}}\;t\;\cdot \;u\;d\Gamma \\ \text{subject}\;\text{to} & g(\rho ) & \equiv \int_{\Omega }\rho \;d\Omega -V_{f}\int_{\Omega }\;d\Omega \leq 0. \end{array} \end{array}$$ (1)

The objective function *f*(*ρ*) represents the compliance, which is the work done by the external forces and is inversely proportional to the structural stiffness. Furthermore, **t** is the load vector, **u** is the displacement vector, and Γ is the boundary of the analysis domain Ω, where Γ_t_ in particular is where the load is applied. The constraint function *g*(*ρ*) limits the amount of available material, and *V*_f_ is the volume fraction of the available material.

We obtained the displacement **u** by solving the following governing equations:

$$\begin{array}{c} \begin{array}{cc} -\nabla \cdot (E:\epsilon (u))=0\;\; & \text{in}\;\;\Omega \\ u=0\;\; & \text{on}\;\;\Gamma_{u}\\ (E:\epsilon (u))\cdot n=t\;\; & \text{on}\;\;\Gamma_{t} \end{array}\}. \end{array}$$ (2)

In the above, ***ϵ*** is the strain tensor $\epsilon \left(u\right)=\frac{1}{2}\left(\nabla u+\nabla u^{⊤}\right)$ and Γ_u_ is the fixed boundary where the displacement is constrained. The linear elastic tensor **E** is expressed as **E** = *ρ*^*P*^ **E**_0_ by the solid isotropic material with penalization method [35]. Furthermore, **E**_0_ is the elastic tensor of the material. The Young’s modulus of the material is 20 GPa and the Poisson’s ratio is 0.3, with reference to the values of zebrafish vertebrae [10, 52, 53]. The positive parameter *P* ≥ 1 enforces the final designs with *ρ*(**x**) at each location being either 0 or 1 through penalization of the stiffness for intermediate densities. In this study, *P* = 3 following [54].

We used the topology optimization method with a time-dependent equation developed by Kawamoto et al. [55] to solve this stiffness maximization problem, because this method is easy to implement using the commercial software COMSOL Multiphysics (we used version 5.4). The details of the equations and parameters are described in S1 Text. For the optimization of multiple load cases, the objective function *f*(*ρ*) was defined with the compliances provided by the different load cases *f*_*i*_(*ρ*) (*i* = 1, 2, …, *N*):

$$\begin{array}{c} f\left(\rho \right)\equiv \sum_{i}^{N}\frac{1}{N}f_{i}\left(\rho \right)=\sum_{i}^{N}\frac{1}{N}\int_{\Gamma_{t_{i}}}\;t_{i}\;\cdot \;u_{i}\;d\Gamma , \end{array}$$ (3)

where *N* denotes the number of load cases. In this study, we used Eq 3 for the optimizations for bending, shear, and torsional loads.

The optimizations in this paper can be reproduced by running S1 Code in LiveLink for MATLAB (we used MATLAB R2018b).

### Analysis domain

**Fig 2:**
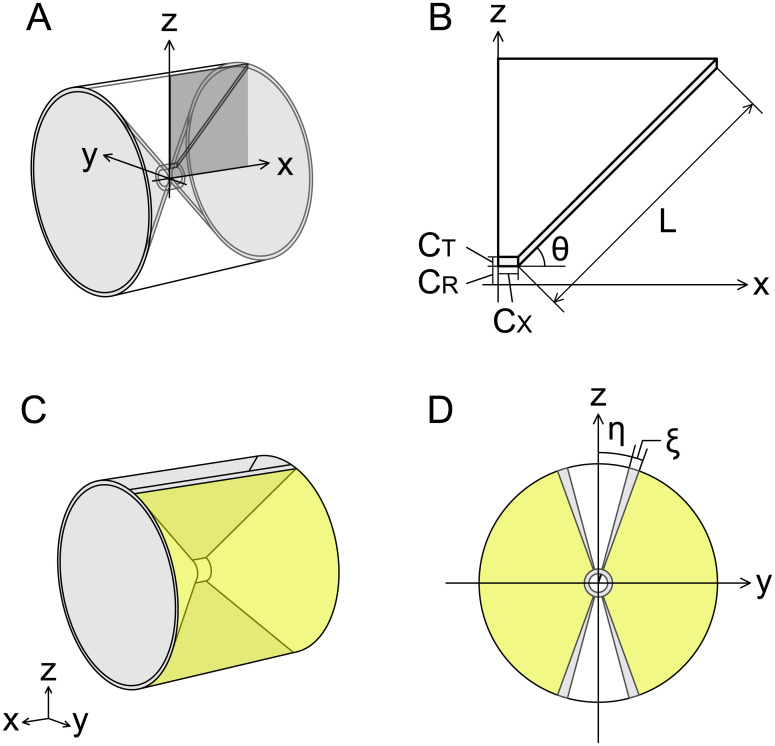
Simulation model. (A) Transparent view of entire structure. The light gray domain corresponds to the autocentrum. (B) Quarter cross-section of analysis domain on zx plane (dark gray plane in (A)). (C) Entire structure. The yellow-colored area is the design domain. (D) Transverse section at midpoint of analysis domain. The light gray wedge-shaped regions represent the vertebral arches. The geometric parameters are described in Table 1.

The analysis domain of our topology optimization model could be divided into the internal and external domains, corresponding to the autocentrum and arcocentrum of teleost vertebral bodies (Fig 2). The internal domain had an amphicoelous hourglass shape, which imitates the autocentrum (Fig 2A). The geometry was defined by the parameters C_R_, C_T_, C_X_, L, and *θ* (Fig 2B). We set the thickness of the hourglass-shaped domain to be small, because the thickness of the autocentrum is very thin in the adult stage. Also, as the angle of conical part of teleost vertebrae ranges approximately from 50° to 90° in many species (S1 Data and S1 Fig), we set the half angle of conical part *θ* to 45°. Because the hourglass-shaped autocentrum and its formation are invariable among teleost species [46, 47, 56], we set the density *ρ* = 1 in the internal domain. Moreover, vertebral arches are formed separately from vertebral bodies during the early developmental stage, which is long before the lateral structure of the vertebral bodies is formed, and are later fused to the vertebral bodies [57, 58]. Based on this process, we set *ρ* = 1 in the domain of the vertebral arches and *ρ* = *d* in the gap of these (Fig 2C and 2D). In this case, *d* is a very small positive approximate value of 0 (see S1 Text), and in this study, *d* = 0.01. The half gap width *η* of vertebral arches was set to 15° because the gap width range was approximately 30° to 60° in many species (S1 Data and S2 Fig). The remaining domain (yellow color in Fig 2) was the design domain in which the distribution of *ρ* was optimized. The geometric parameters are described in Table 1. The analysis domain size was set to approximately 1 cm^3^ considering the size of teleost vertebral bodies.

**Table 1:** Parameter settings to define geometry of analysis domain in Figs 2–6.

Parameter	Symbol	Value
Radius of cylindrical part (chordacentrum)	C_R_	0.04 cm
Thickness of autocentrum	C_T_	0.02 cm
Length of cylindrical part (chordacentrum)	C_X_	0.04 cm
Length of conical part	L	0.6 cm
Half angle of conical part	*θ*	45°
Width of vertebral arches	*ξ*	5°
Half width of gap of vertebral arches	*η*	15°

The initial density value in the design domain was *ρ* = *d*. Therefore, the material was added at the beginning of the optimization process and was removed when the material volume exceeded the upper bound *V*_f_ (S1 Video). In this study, the difference in the initial density values did not influence the optimization results (See S1 Text and S3 Fig).

The optimized structure was displayed with isosurfaces of *ρ* = 0.5.

### Load cases

**Fig 3:**
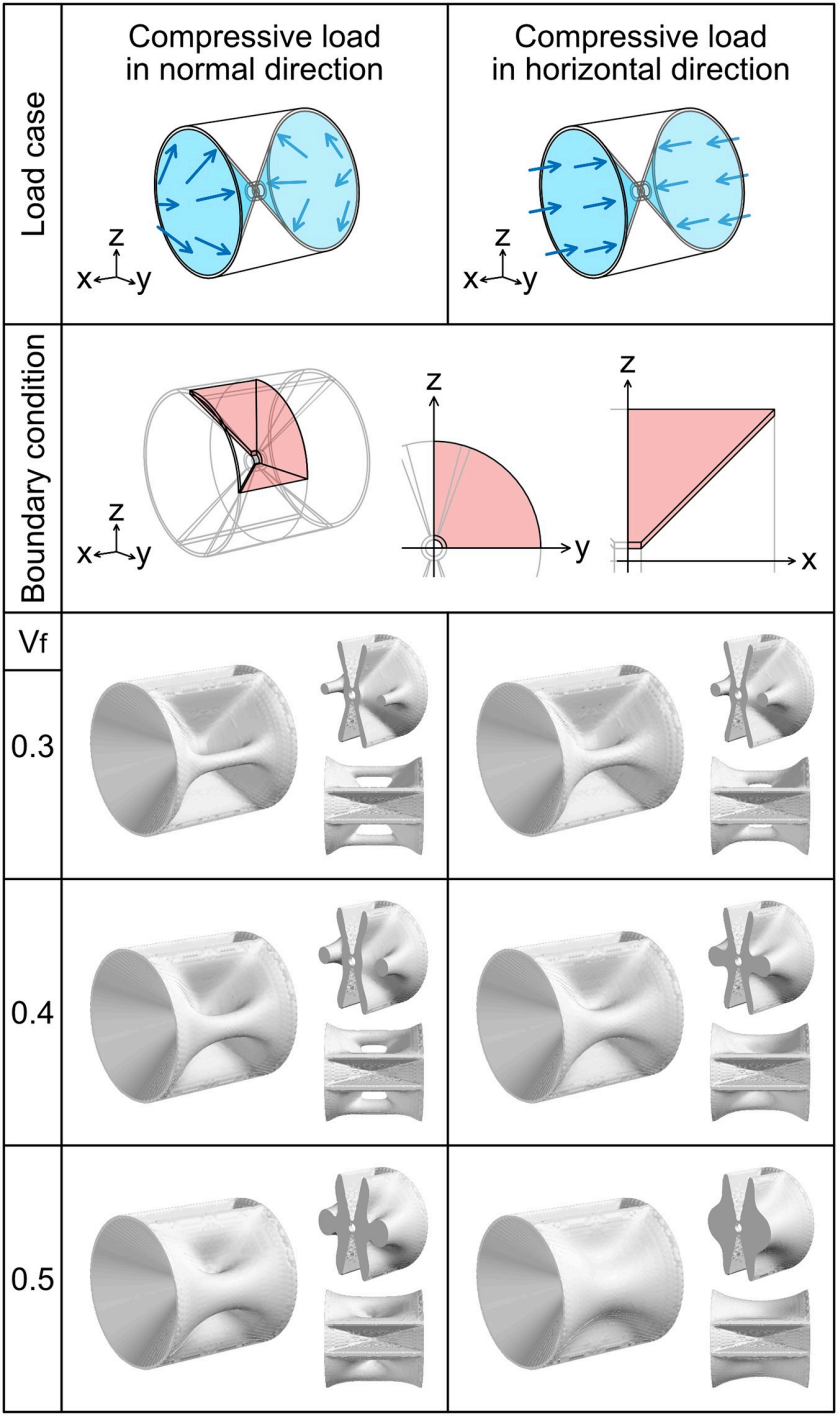
Optimization for compressive loads to autocentrum. The uppermost images are diagrams of the loads. The arrows indicate the load directions. The blue-colored area indicates where the load was applied. The images in the second row from the top show diagrams of the boundary condition; the boundary condition is the same in the two different compressive load cases. We imposed the symmetric boundary condition **u** ⋅ **n** = 0 to the pink-colored area on the xy, yz, and zx planes and used an eighth model (left). The middle and right images in this row indicate the areas to which the symmetric boundary condition was imposed on the yz and zx plane, respectively. The area of the symmetric boundary condition on the xy plane is similar to that on the zx plane. The images in the third, fourth, fifth rows are the entire structures, transverse sections, and top views of the optimization results. The numbers in the leftmost column show the volume fraction.

**Fig 4:**
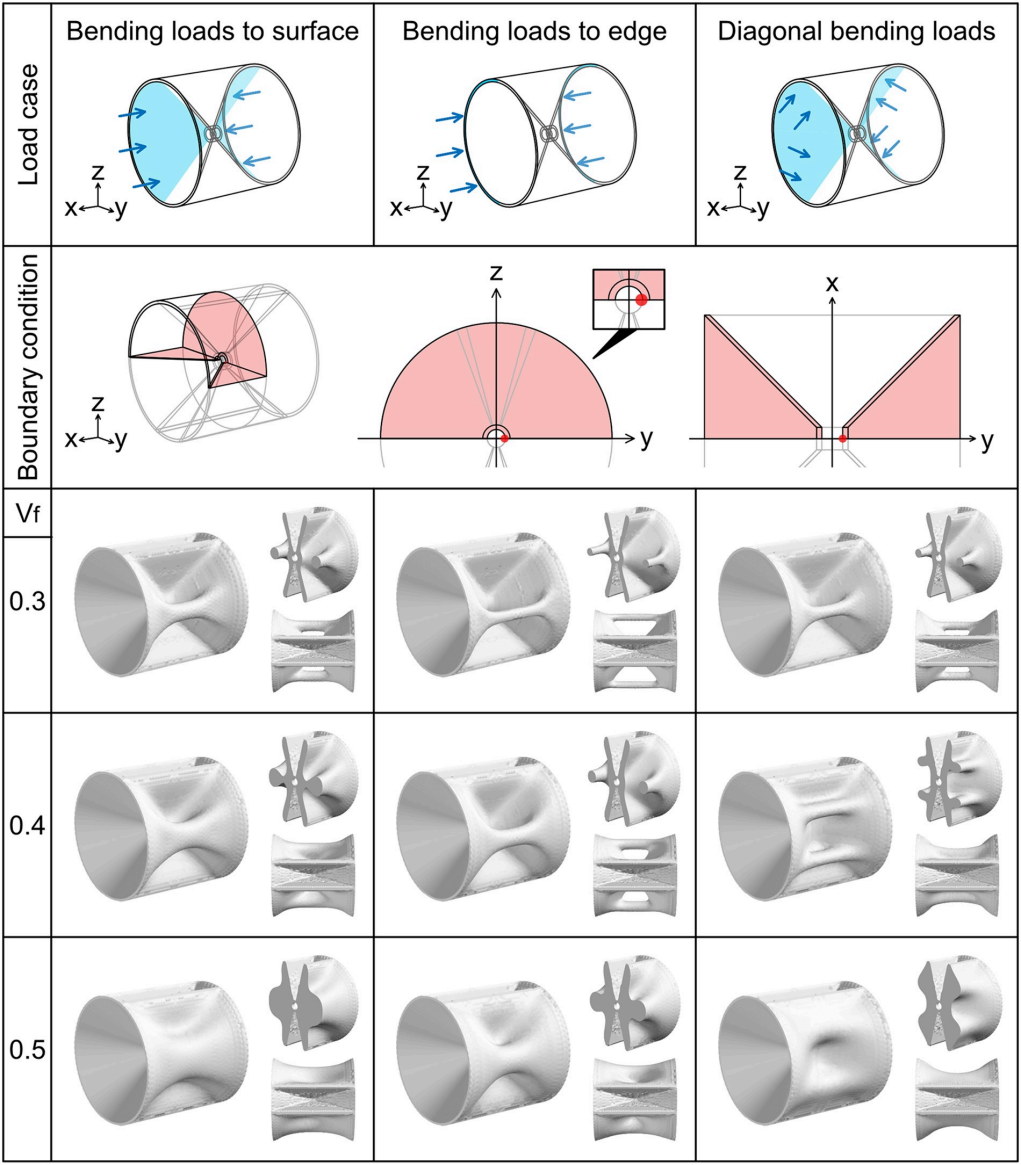
Optimization for bending loads to autocentrum. The arrangement and color scheme of the images are the same as those described in Fig 3. The boundary condition is the same in the three different bending load cases. We imposed the symmetric boundary condition **u** ⋅ **n** = 0 on the xy and yz planes (left image in the second row from the top) and used a quarter model. The middle and right images in the second row from the top are the areas to which the symmetric boundary condition was imposed on the yz and xy plane, respectively. The magnified image of the center on the yz plane is shown. We imposed the displacement constraint *u*_*y*_ = 0 to the point (0, C_R_, 0) (red dot) when the bending loads $t=-F\frac{x}{\left|x\right|}$ were applied to half of the concave surface of the hourglass-shaped domain in *y* ≤ 0 (the blue-colored area). When we applied the bending loads to half of the area in *y* ≥ 0, we imposed the same displacement constraint to the point (0, −C_R_, 0). In optimization for the diagonal bending loads, the results are displayed for *w* = 0.8.

**Fig 5:**
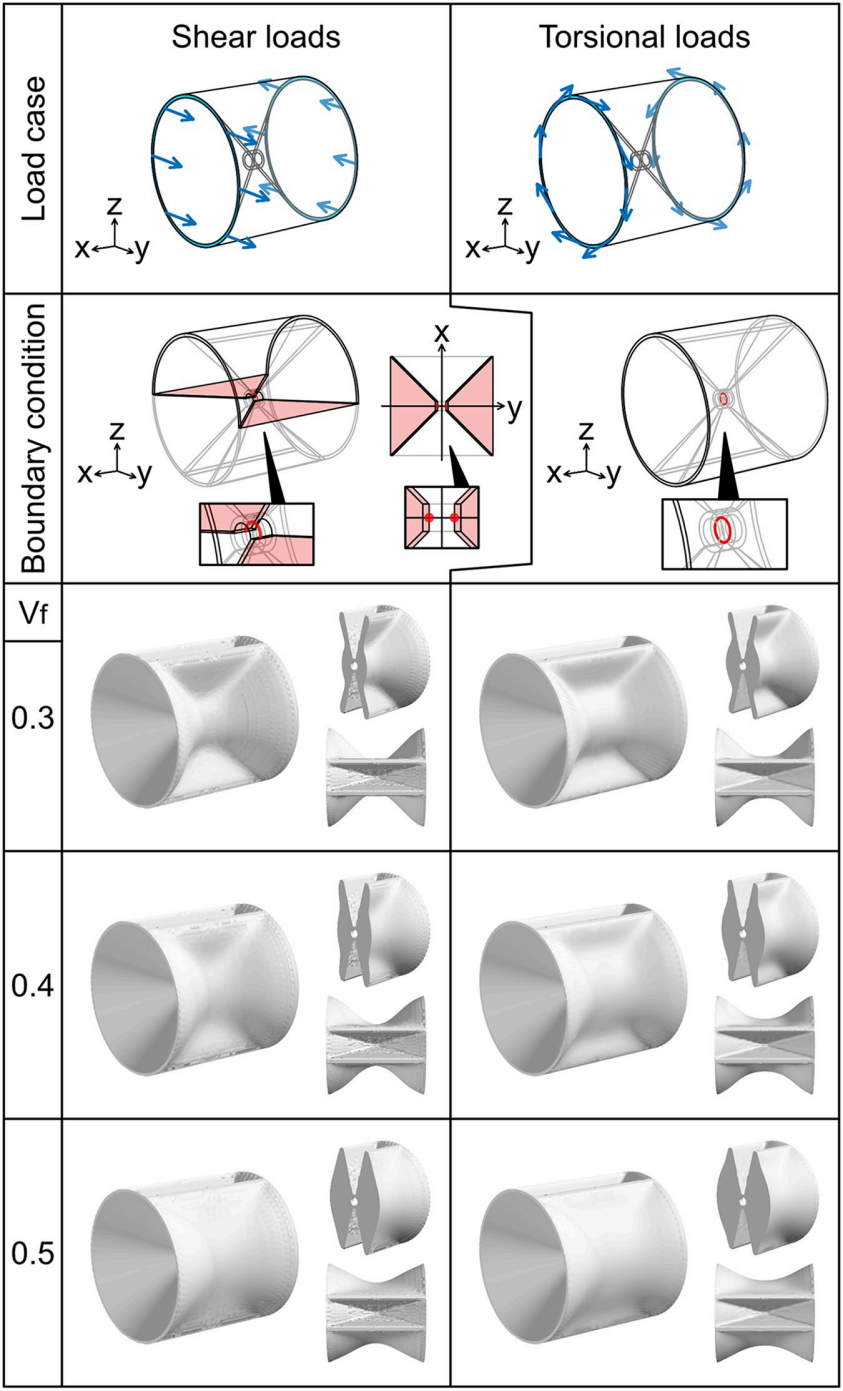
Optimization for shear and torsional loads to autocentrum. The arrangement and color scheme of the images are the same as those described in Fig 3. For the shear loads, we imposed the symmetric boundary condition **u** ⋅ **n** = 0 on the xy plane (left image in the second row from the top) and used a half model. The right diagram is the area to which the symmetric boundary condition is imposed on the xy plane. Magnified images of the center of the analysis domain are shown. We imposed two displacement constraints: *u*_*x*_ = 0 to the points (0, −C_R_, 0) and (0, C_R_, 0) (the red dots), and *u*_*y*_ = 0 to the edge *y*^2^ + *z*^2^ = C_R_^2^ (*z* ≥ 0, the red line). For the torsional loads, we imposed the displacement constraints **u** = 0 to the edge *y*^2^ + *z*^2^ = C_R_^2^ (the red line).

Since fish float in water, the effect of gravity on the teleost vertebrae can be considered to be minimal compared to that on terrestrial vertebrates. Indeed, the vertebrae of the space-flight medaka did not show significant changes in shape and bone mineral density [59]. In addition, a study using an amphibious fish species showed that the bone stiffness of the fish in water was lower than that of the terrestrially acclimated fish, and was comparable to that of fish under simulated microgravity using a random positioning machine [60]. Hence, in this study, we focused on the external loads occurring by deformation of the surrounding tissues. For instance, the lateral muscles are attached to the vertebrae, and the contraction of these muscles for axial body undulation exerts the external load on teleost vertebrae [50, 51]. Also, the intervertebral region between vertebral bodies contains bicone-shaped vacuolated notochord cells [61, 62] and the vertebral bodies are connected on the edges with collagen fiber bundles [63]. Based on these findings, we hypothesized that compressive and bending loads occur on autocentrum. Therefore, we applied compressive and bending loads in multiple directions to the concave surface and edge of the autocentrum (Figs 3 and 4). We also applied shear and torsional loads for the comparison of optimization results (Fig 5).

**Fig 6:**
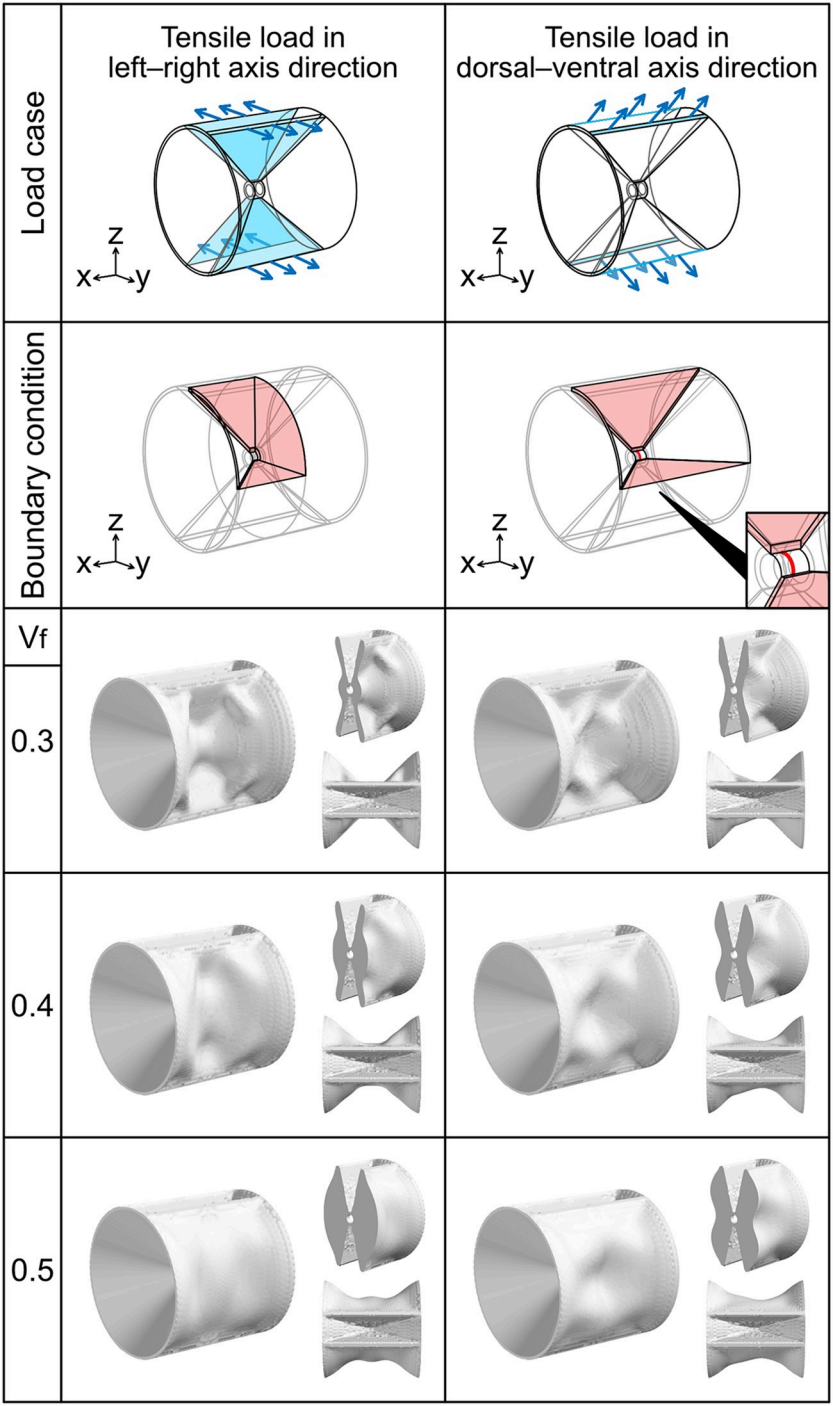
Optimization for tensile loads to vertebral arches. The arrangement and color scheme of the images are the same as those described in Fig 3. For the tensile load in left–right axis direction, we imposed the symmetric boundary condition **u** ⋅ **n** = 0 to the pink-colored areas on the xy, yz, and zx planes and used an eighth model. For the tensile load in dorsal–ventral axis direction, we imposed the symmetric boundary condition to the pink-colored areas on the xy and zx planes and used a quarter model. We also imposed the displacement constraint *u*_*x*_ = 0 to the edge *y*^2^ + *z*^2^ = C_R_^2^ (*y* ≥ 0 and *z* ≥ 0, the red line). The bottom right of the second row shows the magnified image of the center of the analysis domain.

Moreover, as the lateral muscle is attached to the vertebral arches as well as vertebral bodies [51], we assumed that tensile loads to the vertebral arches occurred during body undulation. We applied the tensile loads in the left–right and dorsal–ventral axis directions to the vertebral arches (Fig 6).

The magnitude of each load *F* was 10^6^ N/m^2^ according to the mechanical analyses of teleost bones [64, 65]. In all load cases, we constrained the displacement to avoid the rigid body mode. Furthermore, we imposed the symmetric boundary condition. In the following descriptions, the position vector is **r** = (*x*, *y*, *z*), with **x** = (*x*, 0, 0), **y** = (0, *y*, 0), and **z** = (0, 0, *z*). Moreover, **n** is the normal vector.

The nine types of load cases we attempted were described below:

#### Compressive load in normal direction

A compressive load in the normal direction **t** = −*F***n** was applied to the concave surface of the hourglass-shaped domain (Fig 3).

#### Compressive load in horizontal direction

A compressive load in the horizontal direction $t=-F\frac{x}{\left|x\right|}$ was applied to the concave surface of the hourglass-shaped domain (Fig 3).

#### Bending loads to surface

Optimization was performed for two different bending load cases, which were the bending loads $t=-F\frac{x}{\left|x\right|}$ separately applied to each half of the concave surface of the hourglass-shaped domain (Fig 4).

#### Bending loads to edge

Optimization was performed for two different bending load cases, which were the bending loads $t=-F\frac{x}{\left|x\right|}$ separately applied to each half of the edge of the hourglass-shaped domain (Fig 4).

#### Diagonal bending loads

Optimization was performed for two different load cases, which were the bending loads $t=F(-\frac{x}{\left|x\right|}+w\frac{z}{\left|z\right|})$ separately applied to each half of the concave surface of the hourglass-shaped domain (Fig 4). Here, *w* ≥ 0 was the ratio of the vertical bending load to the horizontal bending load.

#### Shear loads

Optimization was performed for two different load cases: **t** = (0, *F*, 0) was applied to the edge in *x* > 0 and **t** = (0, −*F*, 0) was applied to the edge in *x* < 0 (Fig 5). Another load case was the counter direction of the former load case applied to the abovementioned regions.

#### Torsional loads

Optimization was performed for the clockwise and counterclockwise torsion. For the clockwise torsion, $\text{t}=(0,\frac{F}{\text{R}}z,-\frac{F}{\text{R}}y)\;\left(x>0\right)$ and $t=\frac{F}{R}\left(0,-z,y\right)\;\left(x<0\right)$ were applied to the edge of the autocentrum (Fig 5). R was the radius of the conical part: R = C_T_ + C_R_ + L*sin*(*θ*). For the counterclockwise torsion, the counter direction of the former load case was applied to the abovementioned regions.

#### Tensile load to vertebral arches in left–right axis direction

A tensile load $t=F\frac{y}{\left|y\right|}$ was applied to the external surface of the vertebral arches (Fig 6).

#### Tensile load to vertebral arches in dorsal–ventral axis direction

A tensile load $\text{t}=(-F,0,F\frac{z}{\left|z\right|})$ was applied to the distal edge of the vertebral arches (Fig 6).

## Results

### Optimization for different load cases

We initially attempted optimization for the load cases applied to the vertebral body (Figs 3–5). The optimization result for the compressive load in the normal direction exhibited a pillar structure on the lateral side of the vertebral body. The pillar structure became thicker as *V*_f_ increased. Moreover, the optimization result for the compressive load in the horizontal direction exhibited a pillar structure when *V*_f_ = 0.3, but the position of the pillar was close to the center of the vertebral body. When *V*_f_ = 0.4, the optimization result exhibited a plate-like ridge extending linearly from the center to the distal edge. When *V*_f_ = 0.5, the ridge was thicker and smoother.

The optimization result for the bending loads to the concave surface exhibited a pillar structure when *V*_f_ = 0.3. When *V*_f_ = 0.4, the optimization result had a plate-like ridge, which was thicker in the distal part than in the proximal part of the vertebral body. This structural feature resembled the lateral structure of the vertebral body of *Pagrus major* (Fig 1B, 1B’ and 1B”). When *V*_f_ = 0.5, the ridge was thicker. The optimization result for the bending loads to the edge of the autocentrum exhibited a pillar structure on the lateral side far from the vertebral body center when *V*_f_ = 0.3 and *V*_f_ = 0.4. When *V*_f_ = 0.5, the optimization result exhibited a thick plate-like ridge extending from the vertebral body center to the distal edge. Furthermore, we attempted optimization for the diagonal bending loads with a vertically bending load in addition to the horizontally bending load (Fig 4). We adjusted the ratio of the vertically bending load to the horizontally bending load *w*. When the ratio was small (*w* was 0.1 to 0.6), the optimized structure exhibited a pillar structure. However, when the ratio was *w* = 0.7 and *V*_f_ = 0.4, two pillar structures were formed. When the ratio *w* was 0.8 to 1.0 and *V*_f_ = 0.4, two plate-like ridges were formed (Fig 4). These two plate-like ridges were similar to the lateral structure of the vertebral body of *Zenopsis nebulosa* (Fig 1D, 1D’ and 1D”). When *V*_f_ = 0.5, two thick ridges were formed.

In addition to the reproduction of plate-like ridges, we observed structural variation depending on the amount of available material. The optimization for the horizontal compressive loads or bending loads produced plate-like ridges when *V*_f_ = 0.4 and *V*_f_ = 0.5. However, when *V*_f_ = 0.3, the optimization results exhibited a pillar structure in which the material at the proximal part was removed. In certain teleost species, the lateral structure of the vertebral bodies has internal hollow spaces, in which bone has been removed [49]. Our topology optimization model produced a similar structural feature to the internal hollow spaces.

In the optimization result for the shear loads, the material was added almost uniformly to the hourglass-shaped domain and no pillar or ridge was formed (Fig 5). The optimization result for the torsional loads was similar, but the proximal part of the vertebral body was thicker than that in the optimization result for the shear loads (Fig 5).

We further investigated the optimization for load cases applied to the vertebral arches (Fig 6). The optimization result for the tensile load in the left–right axis direction indicated that four small hump-like ridges were formed on the edges of the vertebral body. When *V*_f_ = 0.4, thicker hump-like ridges were formed (Fig 6). This structural feature was observed in the vertebral body of *Scarus forsteni* (Fig 1F and 1F’). When *V*_f_ = 0.5, the material was added uniformly to the hourglass-shaped domain. Moreover, the optimization result for the tensile load in the dorsal–ventral axis direction along the vertebral arches exhibited four diagonal ridges extending from the vertebral arches to the vertebral body when *V*_f_ = 0.3. These four ridges crossed one another (Fig 6). This structural feature was similar to the tarp-like triangle ridges of the vertebral body of *Macroramphosus sagifue* (Fig 1G and 1G’). When *V*_f_ = 0.4 and *V*_f_ = 0.5, the four ridges were thick.

### Optimization in different analysis domains

To apply our model to the simulation of the external shapes of bones, model validation is an important step. Although we reproduced the morphological features of the teleost vertebrae by setting different load cases, it is difficult to measure the external load condition of real fish. Therefore, we focused on the geometric parameters of the analysis domain. A previous comparative observation of teleost vertebrae [49] showed that geometric parameters such as the aspect ratio of the vertebral bodies and the gap width of the vertebral arches differ among species. In the simulations described above, the vertebral arches and autocentrum were predefined. However, when these geometric parameters change, the calculation results also change. Hence, we can validate our model by comparing the simulation results for different geometric parameters with real teleost vertebrae.

**Fig 7:**
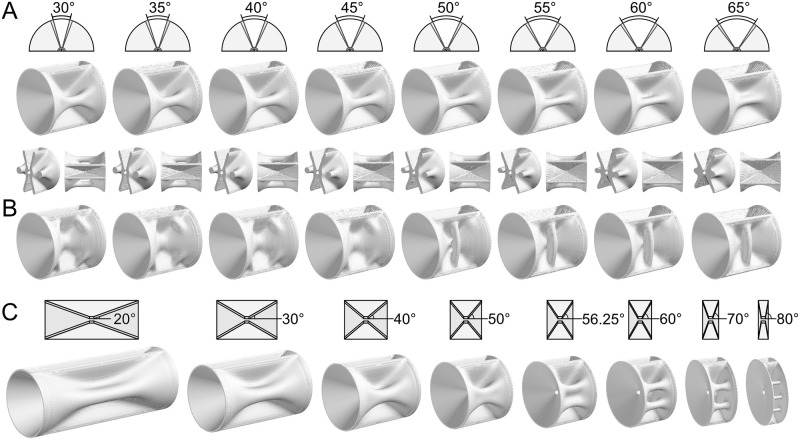
Dependence of lateral structure of vertebral bodies on analysis domain geometry. (A and B) Adjusting the gap width of vertebral arches *η*. The load case was (A) the bending loads to the autocentrum surface in the horizontal direction and (B) the tensile load to the vertebral arches in the left–right axis direction. The optimization results are displayed every five degrees. In (A), the transverse sections and top views are also displayed. (C) Adjusting the vertebral body lengths in the cranial–caudal direction. The value of *θ* is displayed. The volume fraction *V*_f_ is 0.33 in (A), 0.3 in (B), and 0.4 in (C).

In many teleost species, the gap width of the vertebral arches is in the range of 30° to 60° (S2 Fig). Accordingly, we adjusted the half gap width parameter *η* to range from 15° to 32.5° (the gap width was 2*η*). Optimization for the bending loads to the autocentrum surface exhibited a pillar structure when the gap width was relatively small (Fig 7A; see also Fig 4). However, when the gap width was larger at 60°, a thin plate-like ridge was produced. This thin plate-like ridge was observed in the vertebral body of *Acropoma hanedai* (Fig 1C and 1C’). In fact, the gap width of *Acropoma hanedai* is larger than that of *Pagrus Major* (See S1 Data). Note that we compared the gap width of the dorsal arch because the gap width of the ventral arches greatly varies according to the anatomical position. Moreover, we attempted optimization for the tensile load to the vertebral arches in the left–right axis direction. When the gap width was small, the optimization result exhibited hump-like structures on the edges of the vertebral body (Fig 7B). However, when the gap width was 50°, a transverse pillar structure was produced. Moreover, when the gap width was larger than 55°, a transverse plate-like ridge extending from the vertebral body center was produced. A transverse plate-like ridge was observed in the vertebral body of *Muraenesox cinereus* (Fig 1E and 1E’). In fact, the gap width of *Muraenesox cinereus* is larger than that of *Scarus forsteni*. Therefore, the dependence on this parameter matches the relationship between the gap width and the lateral structure of teleost vertebrae.

The autocentrum length in the cranial–caudal direction varies among teleost species [46, 49] (S1 Fig). Because our model could imitate this variation by adjusting the cone angle *θ*, we adjusted *θ* by setting identical vertebral lengths in the dorsal–ventral direction $L\left(\theta \right)=L\frac{\sin\frac{\pi }{4}}{\sin\theta }$ (Fig 7C). The optimization results for the bending loads to the autocentrum surface exhibited a thick plate-like ridge on the lateral side when the vertebral length was large (*θ* was 20 to 40°). When the vertebral length was small, the lateral structure varied from the pillar structure to the divided two-pillar structures (*θ* was 50 to 70°), and when the vertebral length was very small, the divided three-pillar structures were produced (*θ* was 80°). Indeed, the long vertebral bodies of some Perciformes species have no or only one lateral ridge, whereas the short vertebral bodies of some Zeiformes and Pleuronectiformes species have multiple lateral ridges (See Figure 2 of [49]). Based on these simulation results, we verified that our model can demonstrate the variation in the lateral structures of teleost vertebrae.

## Discussion

In this study, we constructed a 3D topology optimization model for the reproduction of teleost vertebrae to test whether the 3D external bone shape can be reproduced using a mathematical model imitating bone formation that depends on external loads. Our topology optimization model produced a variety of 3D structures based on stiffness maximizations for different load cases, and some of these structures exhibited structural features that were similar to the lateral structures of teleost vertebral bodies. This result shows that topology optimization can be applied to reproduce 3D bone external shapes, which can be used to demonstrate the adaptation of bone external shape to external loads. In addition, the variation in lateral structures was reproduced by the difference in load cases and in the shape of the bone parts that are formed independently of external loads. This suggests that our model can be used to explain how adaptation to the external loads and development based on gene expression influence the variation in the shapes of teleost vertebrae.

### Bone internal structure and external shape

Because the internal trabecular bone and the external cortical bone exhibit different structural features, several studies have compared the formation process between these bones [66–68]. However, these bones are similarly formed by osteoblasts and osteoclasts. Furthermore, several studies have reported that the trabecular bones coalesce into the cortical bone in the young growing long bones and jawbones of mammals [69–71]. Based on these facts, some researchers hypothesized that a similar mechanism forms the trabecular and cortical bones. Tanck et al. [72] reproduced the continuous structural changes from the trabecular bones to cortical bones using the bone remodeling algorithm, showing that the thin pillar structures change into thick structures with increasing external loads.

In this study, we reproduced the pillar structure similar to the trabecular bones and the thick plate-like ridges separately, suggesting that the trabecular and cortical bones are indeed formed by a similar mechanism. However, it is difficult to produce a structure with both the thin pillar and thick structural features. In our topology optimization model, the minimum thickness of the structures is regulated by the diffusion term for smoothing the material distribution [55]. We can then adjust the diffusion coefficient to investigate how the extent of bone synthesis and resorption influences bone shape, which relates to the group size of osteoblasts and osteoclasts. To decrease the diffusion coefficient, we need to use the fine mesh that discretizes the design domain. However, simulation with many fine meshes is time-consuming, taking days; thus, fine mesh is not suitable for testing optimizations for different load cases. Therefore, we used the relatively coarse mesh in this study. However, our model could reproduce both the trabecular and cortical bones simultaneously by decreasing the diffusion coefficient and using fine meshes. We previously reported that some species such as tuna have vertebrae in which the internal trabecular bones are covered by the external cortical bones [49]. By reproducing this structure, we can explain the formation of the trabecular and cortical bones according to the variation in the behaviors of osteoblasts and osteoclasts.

### Future improvements to our model

In this study, we used a very simple algorithm in which fewer principles were considered compared with other mathematical models [23, 37, 73] to investigate the dependence of the variation in bone external shapes on load condition. For this reason, our model reproduced only a limited number of the lateral structures of teleost vertebrae. Recent studies have considered more factors for more accurate simulations, such as the intercellular signaling [74], the osteocytic mechanosensory system [75, 76], the site-specific effect on bone formation [77], and the material properties of bones [78–80]. Following these perspectives, our model may be able to reproduce more types of lateral structures by considering additional factors relating to teleost vertebrae.

For instance, we previously found that the lateral structures of most teleost species have equally spaced microcracks extending in the radial direction from the center of the vertebral bodies [49]. This finding suggests that the growth direction of teleost vertebral bodies has a radial anisotropy, which does not depend on external loads. In anglerfish and pufferfish, the lateral structure exhibits a net-like shape that is formed by many thin sheet-like trabeculae extending radially from the center of the vertebral body [49]. Because these species hardly undulate their bodies during swimming [81], we assume that factors other than adaptation to muscle tension influence the formation of the net-like lateral structure. Therefore, introducing anisotropic growth of the vertebrae into our model may allow for reproduction of the net-like structure.

In addition to modifications of the model, we can attempt optimization for different complex load cases and different objectives [82]. For these reasons, topology optimization can be applied to the computer simulations of bone shapes in a variety of vertebrate species. Further studies using computer simulations will help to understand the relationship between bone shapes and external loads.

### Estimating the external loads

Elucidating bone external loads is necessary to understand how bones support the body of vertebrates. Because it is difficult to examine external loads directly, researchers have speculated the external loads based on anatomical observations. For instance, Laerm [46] described that the lateral structure of teleost vertebral bodies adapts to the bending load exerted by fish undulations. This speculation was based on morphological observations of the vertebrae, and it has not been examined. However, our simulation results demonstrated that the bending load is important for formation of the plate-like ridge, thereby supporting Laerm’s speculation. By comparing the optimization results and the teleost vertebrae, we can examine the external loads that are significant to the formation of the teleost vertebrae.

Moreover, other studies have developed methods to calculate the external loads from motion capture [83–86] and from an *ex vivo* loading test [87–89]. We can also utilize these methods to confirm whether our simulation accurately estimates external loads.

Accurate estimation of the external loads will help to improve conventional structural analysis of teleost vertebrae [10, 64], in which the external load condition was speculated based on anatomical observations. Such improved analyses will help to explain the mechanism that maintains the body balance of vertebrates. Implementing the simulations and analyses of the vertebrae in a broader range of teleost species may further help to estimate the locomotion styles of fish.

### Relationship between vertebrae shapes and swimming styles

In teleosts, swimming style greatly varies among species [81]. Previous anatomical studies have shown that according to the swimming styles, species exhibit different material property, morphology, arrangement, and deformation pattern of the lateral muscle and connective tissues [90–95]. These differences in the surrounding tissues can impose different external loads on the vertebrae. In our simulation, the optimizations for the different load cases reproduced the different lateral structures of the vertebral bodies. These findings can lead to a hypothesis that teleost vertebrae adapt these shapes to the external loads determined by swimming styles. If so, we can explain the swimming styles of the extinct species based on the shapes of the vertebrae. Furthermore, we may understand how teleosts have obtained different swimming styles in the phylogenetic history. To confirm this hypothesis, further investigation of whether changes in swimming style can induce changes in vertebrae shape should be performed.

## Materials and methods

### Skeletal specimens of fish

All fish were obtained via commercial bottom trawling in the coast of the Japan archipelago, or purchased from fish markets in Japan. Fish identification followed [96]. To prepare the skeletal specimens, we first boiled the fish for approximately 15 to 30 min, depending on the size of fish, until the body tissues were completely heated. Then, we roughly removed the muscles, and the bones were cleaned by immersion in trypsin solution (trypsin [BECTON DICKINSON Difco Trypsin 250] 1 g in milliQ) for 1 day. We then removed the remaining tissues using running water and air-dried the bones at room temperature (24–25°C).

### Micro-CT scanning

We scanned the skeletal specimens of vertebral bodies from each individual fish using the micro-CT scanner SkyScan 1,172 (SkyScan NV, Aartselaar, Belgium) following the manufacturer’s instructions. For stable positioning, we fixed each specimen to the stage using double-sided tape. The X-ray source was 50 kV, and the datasets were acquired at a resolution of 2.48 to 10.9 μm/pixel, depending on the size of each vertebral body. We reconstructed the transverse section stacks from primary shadow images using the SkyScan software NRecon (Version 1.7.1.0). From these image stacks, we constructed 3D volume-rendered images using the SkyScan software CTVox (Version 3.3.0).

## Supporting information

S1 Text Topology optimization method with time-dependent diffusion equation. (PDF)

S1 Code Code for the simulation. By running this MATLAB script file in LiveLink for MATLAB, we can implement the optimization for compressive load in normal direction. Also, it generates a COMSOL Multiphysics binary file. By editing this binary file, we performed all simulations in this paper. We used COMSOL Multiphysics version 5.4 and MATLAB R2018b.
(M) Click here for additional data file.

S1 Fig Analysis of angle of the conical parts of vertebral body. (A) Method for measuring angle of conical parts. We defined three points to draw lines along the cone and measured the angle (white lines) using the angle tool of ImageJ (https://imagej.nih.gov/ij/). We measured the angles of cranial conical part and caudal conical part. The positions of the vertices at these angles are different in some species because the central part of the vertebral bodies is not exactly straight. (B) Ratio of angle of conical part. We used the vertebral body with the first hemal arch of 32 teleost species. Original measurement data are presented in S1 Data.
(TIFF)

S2 Fig Analysis of gap width of vertebral arches. (A) Method for measuring gap width of vertebral arches. We defined three points to draw lines along the vertebral arch and measured the angle (white lines) using the angle tool of ImageJ (https://imagej.nih.gov/ij/). We measured the angles of dorsal arch and hemal arch. The positions of the vertices at these angles are different in some species because the positions of vertebral arches are not exactly symmetrical. (B) Ratio of gap width of vertebral arches. We used the vertebral body with the first hemal arch of 32 teleost species. Original measurement data are presented in S1 Data.
(TIFF)

S3 Fig Effect of initial density values on optimization results. To investigate the effect of the different initial density values, we adjusted the initial density value in the range of d ≤ *ρ* ≤ 1. The optimization for the diagonal bending loads produced different structures to those shown in Fig 4 when the initial density value in the design domain was *ρ* = 1. When *V*_f_ = 0.4, the result exhibited a pillar structure. In other initial density values, the optimization results were the same as those shown in Fig 4. These different structures are local optima for stiffness maximization, among which the convergent values of the compliance are similar. In this load case, the initial density value influences shape variation. However, the other optimizations performed in this study were not influenced by the initial density values, producing the same structure as those shown in Figs 3–7.
(TIFF)

S1 Data Angle of the conical parts of the vertebral body and gap width of the vertebral arches in 32 teleost species. To obtain these data, we used the micro-CT sections of the vertebrae with the first hemal arch of 32 teleost species. The sections are provided in the SSBD database (http://ssbd.qbic.riken.jp/set/20190301/) (See [49]).
(XLSX)

S1 Video Optimization process for compressive load in normal direction. (GIF)
