# Supplementary material for: Three-dimensional topology optimization model to simulate the external shapes of bone
Source: PLoS Comput Biol. 2021 Jun 16;17(6):e1009043. doi: 10.1371/journal.pcbi.1009043 (PMC8208580; doi:10.1371/journal.pcbi.1009043)
Supplement: S1 Text — (PDF) [file pcbi.1009043.s001.pdf]

Supporting information
Three-dimensional topology optimization model to simulate the external shapes of bone

Misaki Sakashita[a,1], Shintaro Yamasaki[b], Kentaro Yaji[b], Atsushi Kawamoto[c], and Shigeru Kondo[a]

[a]Graduate School of Frontier Biosciences, Osaka University, Suita, Osaka, Japan
[b]Graduate School of Engineering, Osaka University, Suita, Osaka, Japan
[c]Toyota Central R&D Labs., Inc., Nagakute, Aichi, Japan
[1]sakashitamsk@gmail.com

S1 Text

Topology optimization method with time-dependent diffusion equation

To use the time-dependent diffusion equation developed by Kawamoto et al. [1], we expressed the material density function ρ by means of the following regularized Heaviside function of a scalar function ϕ:

$$\rho(\phi) = \begin{cases} d & (\phi < -h); \\ (1-d)\mathrm{H}(\phi, h) + d & (-h \le \phi \le h); \\ 1 & (h < \phi), \end{cases} \tag{1}$$

$$\mathrm{H}(\phi, h) = \frac{1}{2} + \frac{15}{16}\left(\frac{\phi}{h}\right) - \frac{5}{8}\left(\frac{\phi}{h}\right)^3 + \frac{3}{16}\left(\frac{\phi}{h}\right)^5. \tag{2}$$

In this case, $h > 0$ is a parameter for the intermediate bandwidth between the domain full of material (where $h < \phi$) and the void domain (where $\phi < -h$). The bandwidth is equal to $2h$, and d is a very small lower bound that is defined to avoid singularities.

Thereafter, we used the following time-dependent diffusion equation of ϕ:

$$\frac{\partial \phi}{\partial t} = \kappa \nabla^2 \phi - \alpha \widehat{\frac{\mathrm{d}L}{\mathrm{d}\phi}}. \tag{3}$$

$\widehat{\frac{\mathrm{d}L}{\mathrm{d}\phi}}$ is a normalized derivative of the Lagrangian function $L(\rho) \equiv f(\rho) + \lambda g(\rho)$:

$$\widehat{\frac{\mathrm{d}L}{\mathrm{d}\phi}} = \frac{\mathrm{d}f}{\mathrm{d}\phi} \Bigg/ \left\| \frac{\mathrm{d}f}{\mathrm{d}\phi} \right\| + \lambda \frac{\mathrm{d}g}{\mathrm{d}\phi} \Bigg/ \left\| \frac{\mathrm{d}g}{\mathrm{d}\phi} \right\|, \tag{4}$$

where λ is the Lagrange multiplier.

The design sensitivity $\frac{\mathrm{d}f}{\mathrm{d}\phi}$ and $\frac{\mathrm{d}g}{\mathrm{d}\phi}$ are obtained by the chained differentiation, as follows:

$$\frac{\mathrm{d}f}{\mathrm{d}\phi} = \frac{\mathrm{d}f}{\mathrm{d}\rho}\frac{\mathrm{d}\rho}{\mathrm{d}\phi}, \; \frac{\mathrm{d}g}{\mathrm{d}\phi} = \frac{\mathrm{d}g}{\mathrm{d}\rho}\frac{\mathrm{d}\rho}{\mathrm{d}\phi}. \tag{5}$$

Moreover, the norm $\left\| \frac{\mathrm{d}f}{\mathrm{d}\phi} \right\|$ and $\left\| \frac{\mathrm{d}g}{\mathrm{d}\phi} \right\|$ are defined as:

$$\left\| \frac{\mathrm{d}f}{\mathrm{d}\phi} \right\| \equiv \int_\Omega \left| \frac{\mathrm{d}f}{\mathrm{d}\phi} \right| d\Omega \Bigg/ \int_\Omega d\Omega \, , \; \left\| \frac{\mathrm{d}g}{\mathrm{d}\phi} \right\| \equiv \int_\Omega \left| \frac{\mathrm{d}g}{\mathrm{d}\phi} \right| d\Omega \Bigg/ \int_\Omega d\Omega \, . \tag{6}$$

In the simulation, we used tetrahedral elements and set the size to 0.025 cm. We set the time step size to 0.05 s and the time length to 10 s for obtaining the convergent solution. In the optimization for bending loads to the short vertebral body (θ was 80°), we set the time length to 30 s for convergence. Following these, the parameter values were set as described in Table A.

For the simulation of teleost vertebrae, we set the Dirichlet boundary condition $\phi = -1$ on the outer surface of the analysis domain, because teleost vertebrae are surrounded by muscles.

Table A: Parameter settings for numerical computations (*see [1]).

Parameter	Symbol	Value
Half of bandwidth in Eq. (1)	h	1.0
Lower bound for material density	d	0.01
Diffusion coefficient in Eq. (3)	κ	8.0×10^{-7}
Source term coefficient in Eq. (3)	α	1.0
Parameter for updating Lagrange multipliers (*)	β	1.0
Parameter for updating Lagrange multipliers (*)	σ	10^8

We set the initial value of ϕ in the design domain to -1. The initial density value in the design domain was then set to $\rho = d$. Because topology optimization for stiffness maximization gives multiple local optima depending on the initial density values [2], we adjusted the initial value of ϕ in the range of $-1 \leq \phi \leq 1$. When the initial value of ϕ was 1, the optimization results showed the same structure as those produced by setting the initial value of ϕ to -1, except for the optimization for diagonal bending loads (See S3 Fig). However, when we set other initial values such as $\phi = 0$, optimization for diagonal bending loads produced the same structure as obtained when the initial value was $\phi = -1$. Therefore, the optimization results were not greatly influenced by the initial density values in this study.

References

[1] Kawamoto A, Matsumori T, Nomura T, Kondoh T, Yamasaki S, Nishiwaki S. Topology optimization by a time-dependent diffusion equation. International Journal for Numerical Methods in Engineering. 2013;93(8):795–817. doi:10.1002/nme.4407.

[2] Bendsøe MP, Sigmund O. Topology Optimization. Springer-Verlag Berlin Heidelberg; 2004. doi:10.1007/978-3-662-05086-6.
