# Supplementary figures and images for: Three-dimensional topology optimization model to simulate the external shapes of bone
Source: PLoS Comput Biol. 2021 Jun 16;17(6):e1009043. doi: 10.1371/journal.pcbi.1009043 (PMC8208580; doi:10.1371/journal.pcbi.1009043)

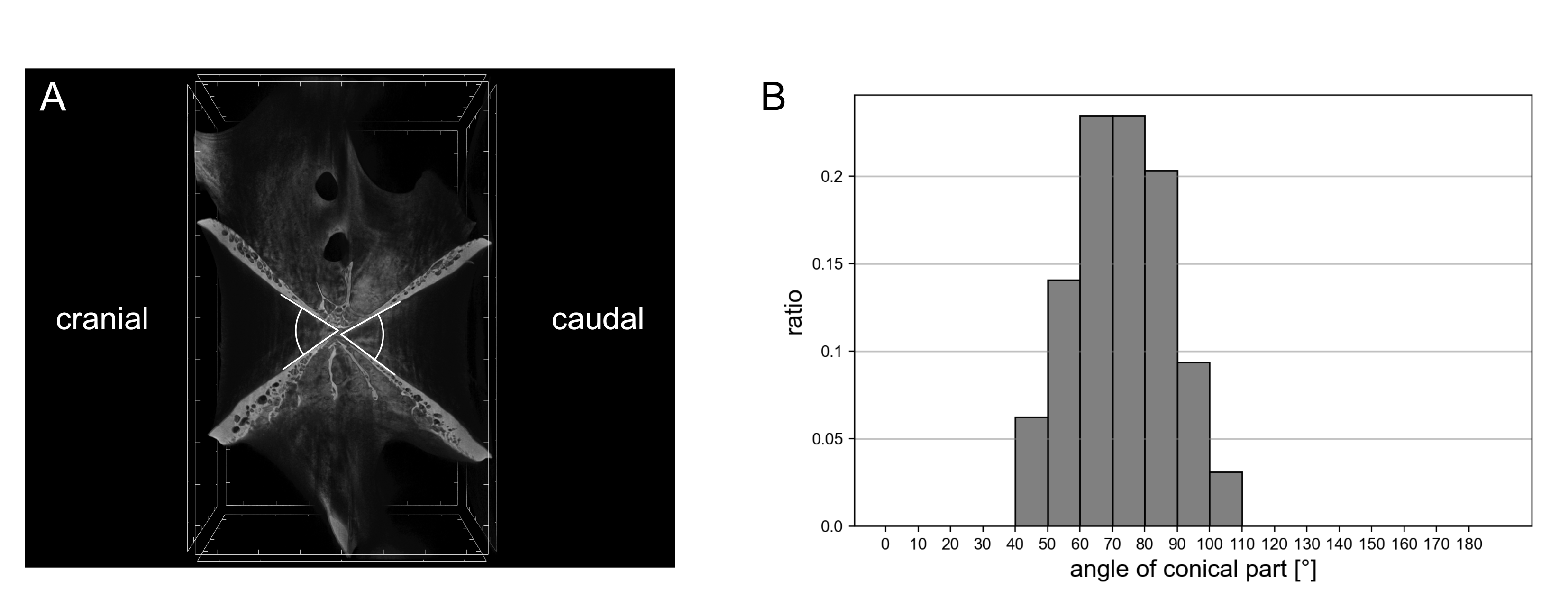

Supplement: S1 Fig — (A) Method for measuring angle of conical parts. We defined three points to draw lines along the cone and measured the angle (white lines) using the angle tool of ImageJ (https://imagej.nih.gov/ij/). We measured the angles of cranial conical part and caudal conical part. The positions of the vertices at these angles are different in some species because the central part of the vertebral bodies is not exactly straight. (B) Ratio of angle of conical part. We used the vertebral body with the first hemal arch of 32 teleost species. Original measurement data are presented in S1 Data. (TIFF) [file pcbi.1009043.s003.tiff]

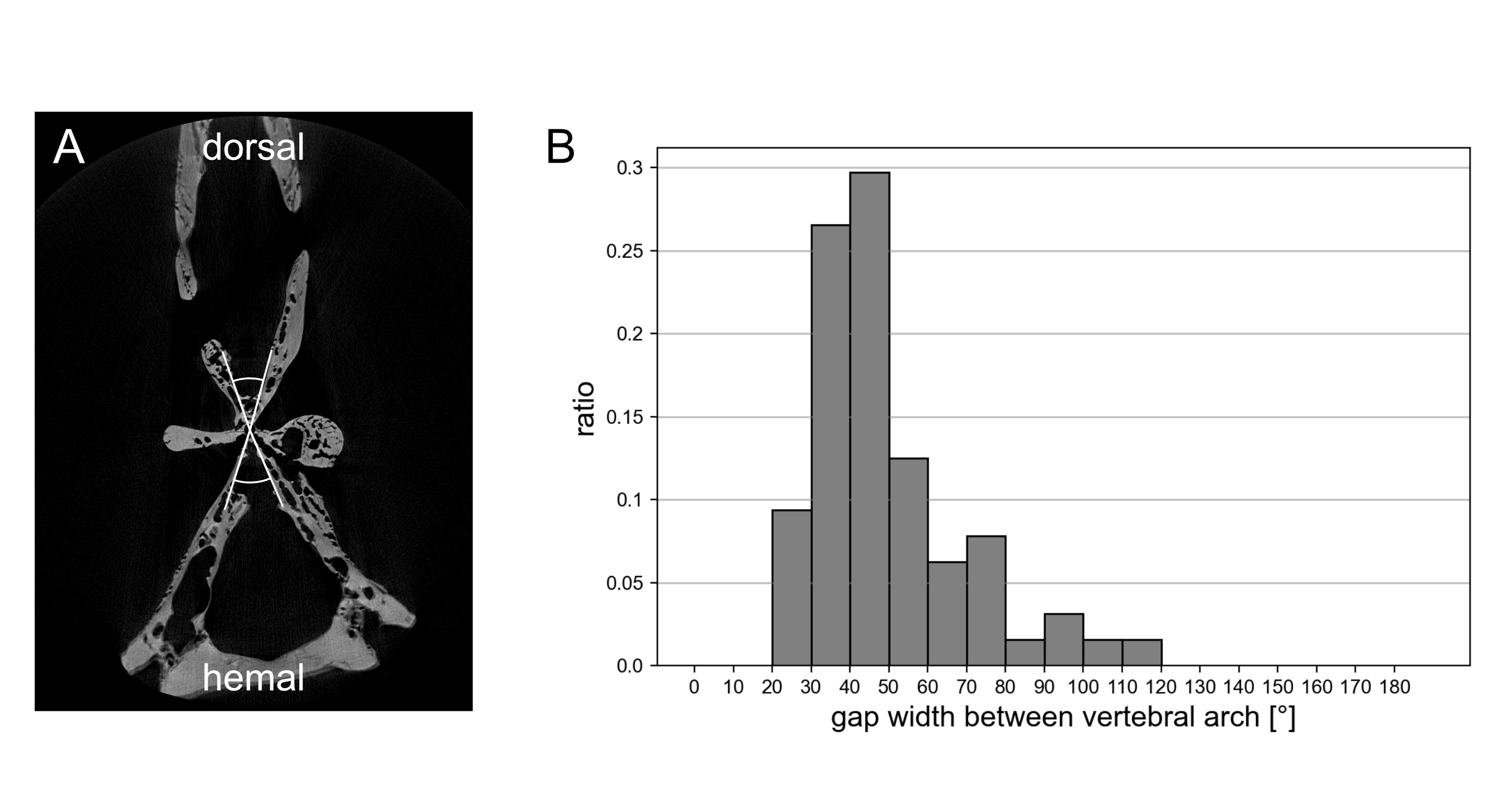

Supplement: S2 Fig — (A) Method for measuring gap width of vertebral arches. We defined three points to draw lines along the vertebral arch and measured the angle (white lines) using the angle tool of ImageJ (https://imagej.nih.gov/ij/). We measured the angles of dorsal arch and hemal arch. The positions of the vertices at these angles are different in some species because the positions of vertebral arches are not exactly symmetrical. (B) Ratio of gap width of vertebral arches. We used the vertebral body with the first hemal arch of 32 teleost species. Original measurement data are presented in S1 Data. (TIFF) [file pcbi.1009043.s004.tiff]

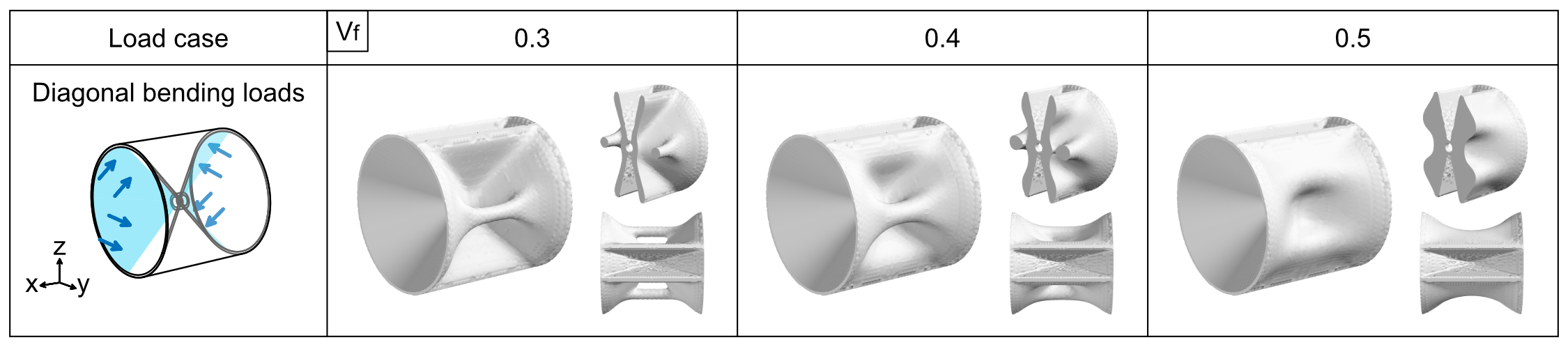

Supplement: S3 Fig — To investigate the effect of the different initial density values, we adjusted the initial density value in the range of d ≤ ρ ≤ 1. The optimization for the diagonal bending loads produced different structures to those shown in Fig 4 when the initial density value in the design domain was ρ = 1. When Vf = 0.4, the result exhibited a pillar structure. In other initial density values, the optimization results were the same as those shown in Fig 4. These different structures are local optima for stiffness maximization, among which the convergent values of the compliance are similar. In this load case, the initial density value influences shape variation. However, the other optimizations performed in this study were not influenced by the initial density values, producing the same structure as those shown in Figs 3–7. (TIFF) [file pcbi.1009043.s005.tiff]

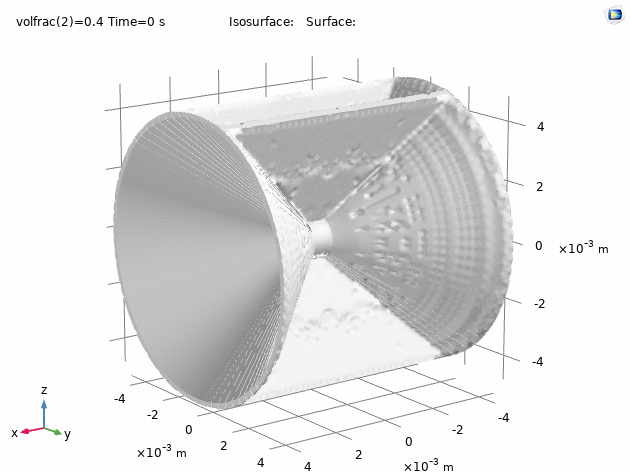

Supplement: S1 Video — (GIF) [file pcbi.1009043.s007.gif]
